# First records of powdery mildew fungi (Erysiphales) on medicinal plants in Taiwan

**DOI:** 10.1186/s40529-020-00307-0

**Published:** 2021-01-06

**Authors:** Yu-Wei Yeh, Pei-Yi Chou, Hsin-Yu Hou, Roland Kirschner

**Affiliations:** 1 School of Forestry & Resource Conservation, National Taiwan University, Taipei, Taiwan; 2 Department of Clinical Pathology, Cheng Hsin General Hospital, Taipei, Taiwan

## Abstract

**Background:**

Production of medicinal plants in Taiwan is not only hampered by international market competition, but also lack of knowledge of their pathogens, such as powdery mildew fungi (Erysiphales, Ascomycota). Records of these fungi in Taiwan originate from few researchers for the last one hundred years and are still incomplete. Since powdery mildews in tropical/subtropical environments rarely develop the sexual stages with morphologically diagnostic characteristics, internal transcribed spacer sequences (ITS) of the ribosomal RNA genes obtained from the asexual stages have become important modern tools for species identification.

**Results:**

Powdery mildews on medicinal plants from educational and ornamental plantations in Taiwan were identified based on the anamorph morphology and ITS sequences. Four powdery mildews on medicinal plants are new records for Taiwan, *Arthrocladiella mougeotii* on *Lycium chinense*, *Erysiphe glycines* on *Pueraria lobata*, *Erysiphe lespedezae* on *Bauhinia* sp., *Desmodium caudatum*, and *Uraria crinita*, and *E. lonicerae* on *Lonicera japonica*. *Eryngium foetidum* is a new host for *Erysiphe heraclei* hitherto known on other host plants in Taiwan. *Eryngium foetidum* and *Uraria crinita* are new host plants for powdery mildews worldwide. Only specific field collection of the pathogens yielded the new records, not checking plant specimens in a phanerogam herbarium. The pathogens did not cause death of the host plants, but appeared to enhance stress by infection of mature leaves.

**Conclusions:**

Taxonomic study of powdery mildews in Taiwan results into new host records of economically important medicinal plants in Taiwan with potential consequences for plant production and quarantine and also shows that host records are quite incomplete worldwide. Although ITS sequences were useful for species identification, the lack of data for several species on the same host genus on the one hand and the low variation between closely related species on the other indicate the need for further study.

## Background

For enhancing the domestic market as well as export of agricultural products from Taiwan, the Council of Agriculture of Taiwan (COA) has established the Good Agricultural Practices (GAP) and the COA-certified organic labeling system (https://eng.coa.gov.tw). Particularly for trading products based on medicinal plants, the GAP system is an important competitive advantage compared to cheaper products from outside Taiwan. *Lycium chinense* is one of the twenty most important medicinal plants covered by the GAP system, but the domestic production is scarce compared to the import from mainland China (Taiwan Institute of Economic Research 2017, http://www.biotaiwan.org.tw/download/structure4/%E5%8A%89%E4%BE%9D%E8%93%81/107/%E6%88%91%E5%9C%8B%E4%B8%AD%E8%8D%89%E8%97%A5%E7%94%9F%E7%94%A2%E5%8F%8A%E9%80%B2%E5%8F%A3%E6%A6%82%E6%B3%81%20(201712).pdf). An important requirement in the COA/GAP certification is the transparent and reduced usage of pesticides. The COA also promotes the development of recreational farms and forests for tourism where medicinal plants and other crops are produced for demonstration and local distribution rather than for mass production. In these environments, pesticides are usually much more rarely used than in industrial agriculture. Under these conditions, pathogens may spread more easily so that knowledge of their occurrence is the first prerequisite for estimating potential risks and for control measures.

Powdery mildew fungi (Erysiphales) are obligate biotrophic plant pathogens which form conspicuous white mycelia on leaves, stems and fruits of specific angiosperm host plants (Braun and Cook 2012). In addition to the mycelium and anamorph features causing the symptoms, sexually formed ascomata provide morphological clues for species identification. Under tropical/subtropical conditions, however, instead of rarely formed ascomata, asexual structures such as mycelial appressoria, conidiophores and conidia of the anamorph stage provide the single morphological features which are less diagnostic for morphological species identification (Piepenbring et al. 2011). Under such conditions, sequences of the internal transcribed spacer (ITS) of the ribosomal RNA genes have become an important tool for species identification (Takamatsu et al. 2015a, b, Meeboon and Takamatsu 2016, 2017a, b, 2020; Khodaparast et al. 2019). Identification and documentation of Erysiphales in Taiwan started over one hundred years ago, followed by occasional records of few other mycologists and plant pathologists as compiled by Kuo (1998) and subsequent publications (Kirschner and Chen 2008; Kirschner and Liu 2014; Chen and Kirschner 2018; Yeh et al. 2018; Kirschner et al. 2020).

On some medicinal plants grown in a botanical garden, recreation and educational farms and as ornamentals, we found powdery mildews hitherto unrecorded for these plants worldwide or in Taiwan. The aim of this study was to identify and document the fungal species on their hosts, based on fresh field collection as well as on dried plant herbarium specimens.

## Methods

### Sampling and morphology

Plant specimens with powdery mildew symptoms were collected in educational and tourist gardens or farms as well as a university campus and kept at ca. 8 °C before study. For light microscopical study, fresh specimens were removed from the leaf surface with transparent tape, mounted in 5–10% KOH and observed at 1000× magnification. For presenting sizes, length and width, 30 structures were measured (if not stated otherwise) and given in the descriptions as mean value ± standard deviation with extreme values in brackets. Drawings were made free hand using scaled paper. Specimens were dried by pressing plant samples between paper and subsequent final drying on an electrical dryer. Representative specimens were deposited at the National Museum of Natural Science, Taichung, Taiwan (TNM). For evaluating the presence of powdery mildews on plant herbarium specimens, we checked 7 specimens of *Eryngium foetidum*, more than 30 of *Lonicera japonica*, 9 of *Lycium chinense*, and over 50 of *U. crinita*, all collected in Taiwan between the years 1917 and 2004 and kept in the herbarium of National Taiwan University (TAI). In cases where colonies of powdery mildew were assumed to be present on leaves, they were removed with a transparent tape and transferred to a lactophenol/cotton blue mounting for microscopical observation.

### Molecular identification

**Fig. 1:**
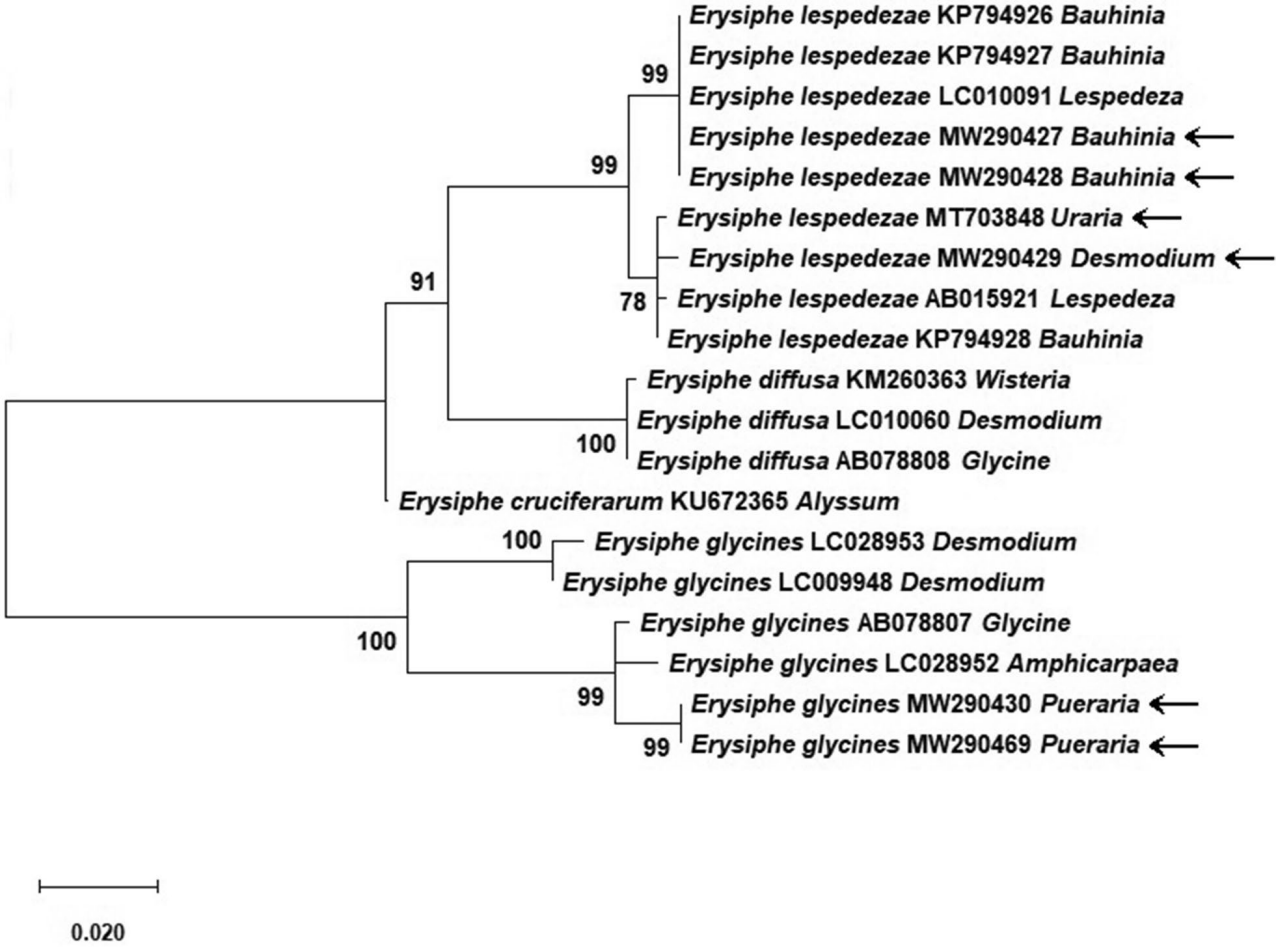
Maximum likelihood analysis of ITS sequences of selected Erysiphales indicating the identification of specimens on medicinally used Fabaceae from Taiwan. Bootstrap values of 1000 replicates above 50% shown. Scientific name of powdery mildew followed by GenBank number and host genus name (new sequences indicated with arrow)

For molecular identification with internal transcribed spacer (ITS) sequences of the nuclear ribosomal RNA genes (ITS 1, 5.8S rDNA, ITS2 and bordering short fragments of the 18S and 28S rDNA), total genomic DNA was isolated from freshly collected mycelium, conidiophores and conidia; the ITS was amplified, sequenced, and edited as in Wei and Kirschner (2017). ITS sequences were used for megaBLAST searches at GenBank and deposited in GenBank. Sequences from BLAST searches and Meeboon and Takamatus (2017a) were selected for confirming the megaBLAST identification of mildews on Fabaceae also with phylogenetic analyses. A phylogenetic estimate for the powdery mildew specimens from Fabaceae was based on an alignment made in MEGA7 with the default options of MUSCLE (Kumar et al. 2016). Except for trimming the ends of the alignment block, no further manipulations were done. The alignment was analyzed with the Maximum Likelihood method with the Kimura-2 parameter model (gamma-distributed) as best model and 1000 bootstrap replicates (Kumar et al. 2016). GenBank numbers and host names are given in Fig. 1.

## Results

Five species of powdery mildews were found only in the anamorph stage on mature leaves of the host plants. The ITS sequences obtained in this study ranged between 570 and 680 bp. Based on anamorph morphology, host specificity, and identification with BLAST as well as ML analysis, four powdery mildews on medicinal plants are new records for Taiwan, *Arthrocladiella mougeotii* on *Lycium chinense*, *Erysiphe glycines* on *Pueraria lobata*, *E. lespedezae* on *Bauhinia* sp., *Desmodium caudatum*, and *Uraria crinita*, and *E. lonicerae* on *Lonicera japonica*. *Eryngium foetidum* is a new host for *Erysiphe heraclei* hitherto known on other host plants in Taiwan. *Eryngium foetidum* and *Uraria crinita* are new host plants for powdery mildews worldwide. The phylogenetic analysis of the powdery mildews on Fabaceae identified in this study confirmed the species identification, but also indicated well supported subclades in *E. glycines* and *E. lespedezae* (Fig. 1). The pathogens did not cause death of the host plants, but appeared to enhance stress by colonizing mature leaves. The fungi were only found on specifically mycologically collected specimens in the field, but not among over 80 plant herbarium specimens collected during the last 100 years in Taiwan. A documentation of the morphological and molecular identification of the five powdery mildew species is given below.

### *Arthrocladiella mougeotii* (Lév.) Vassilkov Fig. 2

*Colonies amphigenous on leaves* Hyphae smooth, 5–6 μm wide. Hyphal appressoria mostly solitary, nipple-shaped. Conidiophores arising solitarily from middle of hyphal mother cell, slightly constricted at base, composed of foot cell and 1–4 distal cells, (53–)62–89(–109) × (8–)9–11 μm. Foot cell with basal septum at same level as upper surface of supporting hypha, straight or curved, (33–)37–52(–60) × (8–)9–11 μm. Conidia catenate, ellipsoid-ovoid to doliiform, (25–)27–32(–35) × (10–)11–13(–15) μm, germination by forming long hyphae close to one of both ends of the conidium, or short hyphae at one or both ends and terminating into nipple-shaped appressoria.

**Fig. 2:**
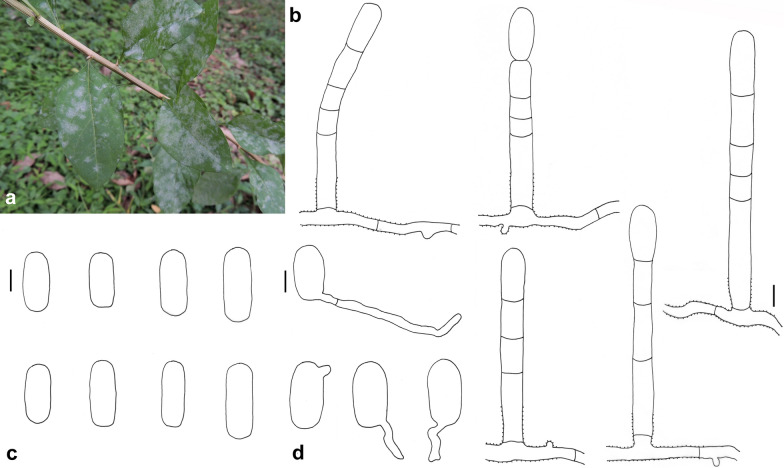
*Arthrocladiella mougeotii* on *Lycium chinense* (AKII 0082, except in **a**. **a** Powdery mildew symptoms on leaves (Taipei, National Taiwan University campus, 23 April 2015). **b** Hyphae with nipple-shaped appressoria and conidiophores. **c** Conidia. **d** Conidia with germination hyphae. Scale bars = 10 μm

*Specimens examined* On leaves of *Lycium chinense* Mill. (Solanaceae). Tainan City, Danei District, Zoumalai Recreation Farm, ca. 23.134596, 120.428836, ca. 740 m, 09 Apr 2008, R. Kirschner 3151 (TNM); Taipei City, Zhongzheng District, sidewalk near MRT Taipower Building Station, ca. 25.020751, 121.528368, ca. 15 m, 27 Apr 2020, AKII 0082 (TNM), ITS sequence GenBank MT703830; Taipei City, Daan District, Taipei, National Taiwan University campus, Zhoushan Road, 23 Apr 2015, not preserved (Fig. 2a).

*Notes* The ITS sequence was 100% identical to over ten published ones exceeding 590 bp of *Arthrocladiella mougeotii* in GenBank. *Arthrocladiella mougeotii* is the single species of its genus and is almost globally distributed and limited to *Lycium* hosts (Braun and Cook 2012). In contrast to Farr and Rossman (2020) who list several powdery mildew species for *Lycium* species, only *A. mougeotii* and *Phyllactinia chubutiana* Havryl., S. Takam. & U. Braun are accepted for *Lycium* hosts in Braun and Cook (2012). Both fungi can be distinguished not only by different ITS sequences, but also the solitary conidia in *Ph. chubutiana* and catenate conidia in *A. mougeotii*.

### *Erysiphe glycines* F. L. Tai on *Pueraria lobata* Fig. 3

*Colonies amphigenous on leaves* Hyphae smooth, 4–6 μm wide. Hyphal appressoria solitary or in opposite pairs, nipple-shaped or lobed. Conidiophores arising solitarily from middle of hyphal mother cell, slightly rough at base, composed of foot cell and 1–4 distal cells. Foot cell with basal septum at same level as upper surface of supporting hypha, straight or curved, (16–)20–30(–34) × (7–)8–10(–11) μm. Conidia solitary, ellipsoid-ovoid to doliiform, smooth-walled, (27–)31–39(–42) × 16–21(–24) μm, germination not found.

**Fig. 3:**
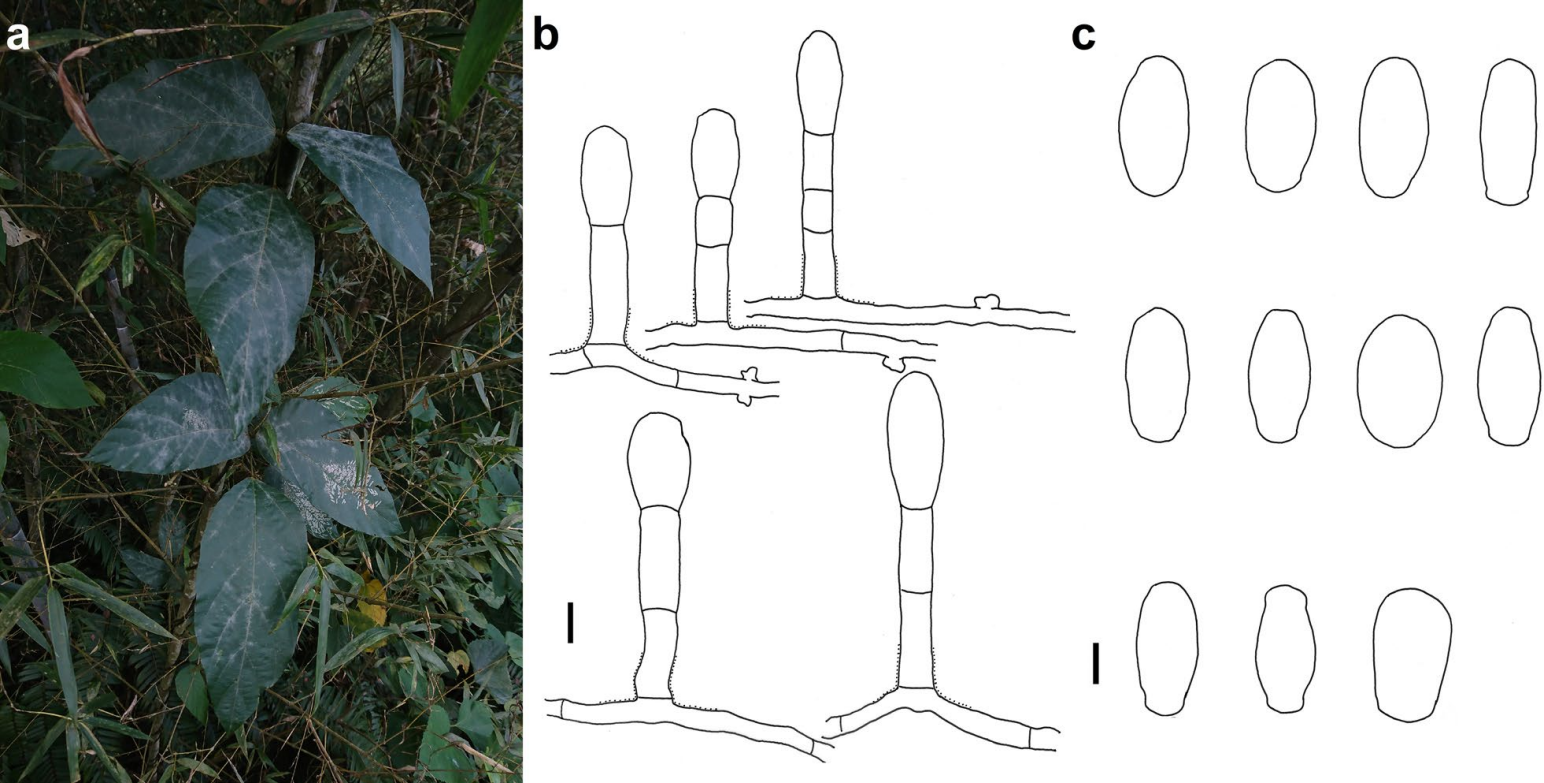
*Erysiphe glycines* on *Pueraria lobata* (R. Kirschner 4638). **a** Powdery mildew symptoms on leaves. **b** Hyphae with appressoria and conidiophores with curved foot cell. **c** Conidia. Scale bars = 10 μm

*Specimens examined* On leaves of *Pueraria lobata* (Willd.) Ohwi (Fabaceae). Hsinchu City, Wufeng Township, Zhulin Village, ca. 24.620047, 121.096907, ca. 680 m, 18 May 2018, R. Kirschner 4638 (TNM), ITS sequence GenBank MW290430; Nantou County, Lugu Township, National Taiwan University Experimental Forest, Fenghuang Nature Education Area, ca. 23.729412, 120.790430, ca. 800 m, 7 Feb 2020, R. Kirschner 4875 (TNM), ITS sequence GenBank MW290469; same place, 20 Oct 2020, R. Kirschner 5068 (TNM).

*Notes* When ITS sequences exceeding 600 bp were compared, there was 99% identity with 6 different bp between our specimen and five published specimens labeled as *E. glycines* in GenBank. Besides there are another four published specimens also labeled as *E. glycines* with 94% identity. In *E. glycines*, the sequences on *Pueraria lobata* form a well-supported subclade, which may represent *E. puerariae* R.Y. Zheng & G.Q. Chen, hitherto only known from *P. lobata* in mainland China for which hitherto no anamorph or DNA data are available.

### *Erysiphe heraclei* DC. Fig. 4

*Colonies amphigenous on leaves* Hyphae smooth, 4–7 μm wide. Hyphal appressoria solitary or in opposite pairs, nipple-shaped or lobed. Conidiophores arising solitarily from middle of hyphal mother cell, slightly curved at base, (56–)68–105(–117) × (6–)7–8(–9) μm. Foot cell with basal septum at same level as upper surface of supporting hypha or slightly elevated for up to 9 μm, straight or curved, (32–)38–49(–52) × (6–)7–8(–9) μm, followed by 1–4 cells. Conidia solitary, ellipsoid-ovoid to cylindrical, (34–)35–45(–51) × (11–)12–17(–19) μm, germination from one of both ends by forming long hypha with nipple-shaped or lobed appressoria.

**Fig. 4:**
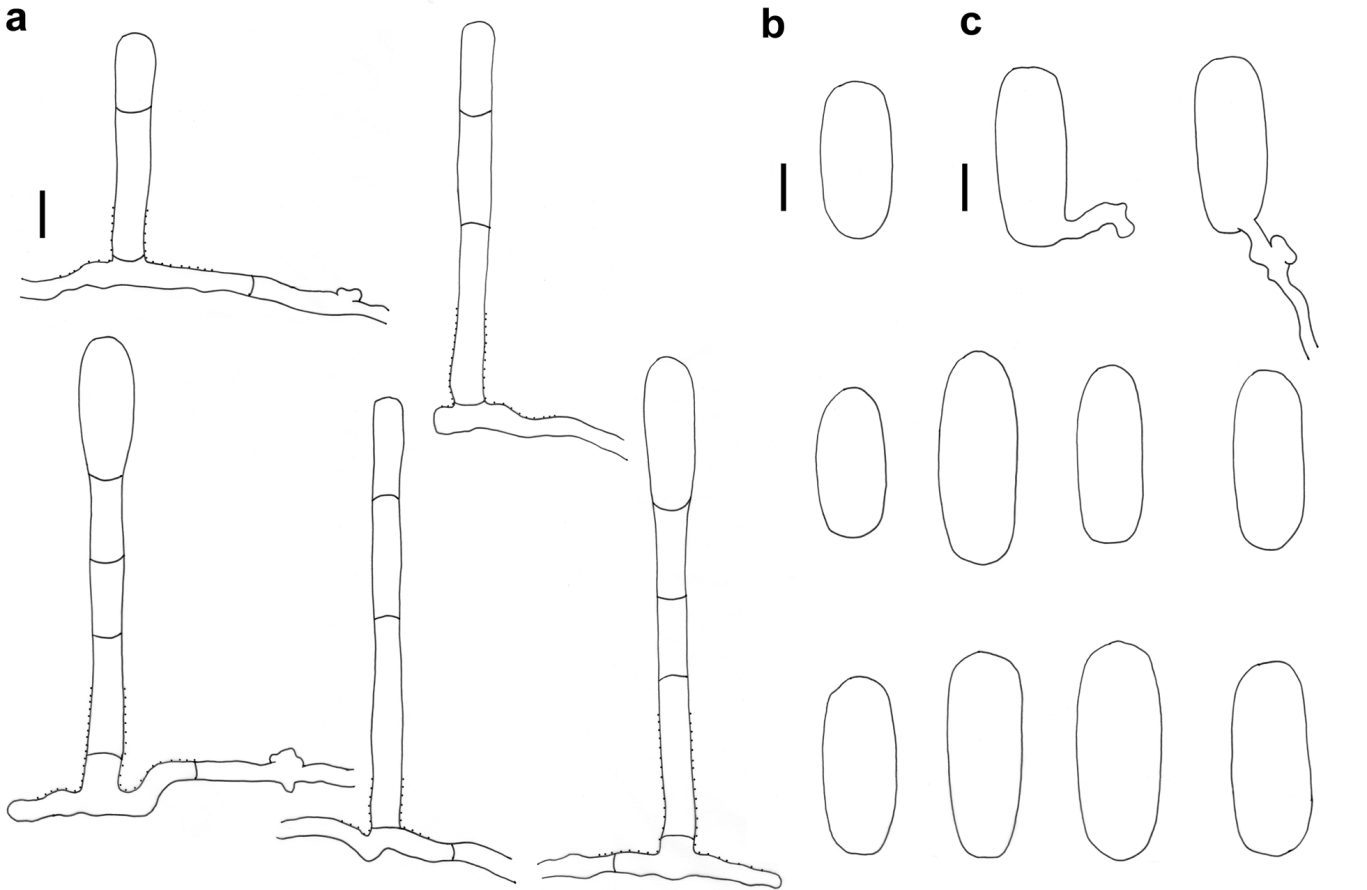
*Erysiphe heraclei* on *Eryngium foetidum* (R. Kirschner 4729). **a** Hyphae with lobed appressoria and conidiophores. **b** Conidia. **c** Conidia with lobed appressoria on germination hyphae. Scale bars = 10 μm

*Specimen examined* On leaves of *Eryngium foetidum* L. (Apiaceae). Taipei City, Daan District, Changhsin Street, National Taiwan University dormitories, ca. 25.015449, 121.546735, ca. 20 m, 24 May 2019, R. Kirschner 4729 (TNM), ITS sequence GenBank MT703849.

*Notes* There was 99% ITS sequence identity with 1 to 6 different bp between our specimen and over ten other published specimens labeled as *E. heraclei* in GenBank. Presently, it is not possible to distinguish between *E. betae* (Vaňha) Weltzien, *E. heraclei*, and *E. malvae* V.P. Heluta based on rDNA sequences (Takamatsu et al. 2015a). The three species are hardly distinguishable also in morphology (Braun and Cook 2012). Future study may reveal whether the species can be separated with additional markers or need to be put into synonymy. In this case, *E. heraclei* would have priority since it is the oldest available name.

### *Erysiphe lespedezae* R.Y. Zheng & U. Braun on *Bauhinia* sp. Fig. 5

*Colonies amphigenous on leaves and petioles* Hyphae smooth, 4–5 μm wide. Hyphal appressoria solitary or in opposite pairs, nipple-shaped or lobed. Conidiophores arising solitarily from middle of hyphal mother cell, mostly curved and slightly rough at base, composed of foot cell and 1–4 distal cells, (59–)70–92(–105) × (4–)5–6 μm. Foot cell with basal septum at same level as upper surface of supporting hypha, cylindrical, mostly curved, (33–)46–68(–80) × (4–)5–6 μm. Conidia solitary, ellipsoid-ovoid to doliiform, (25–)29–41(–49) × (11–)12–16(–18) μm, germination not found.

**Fig. 5:**
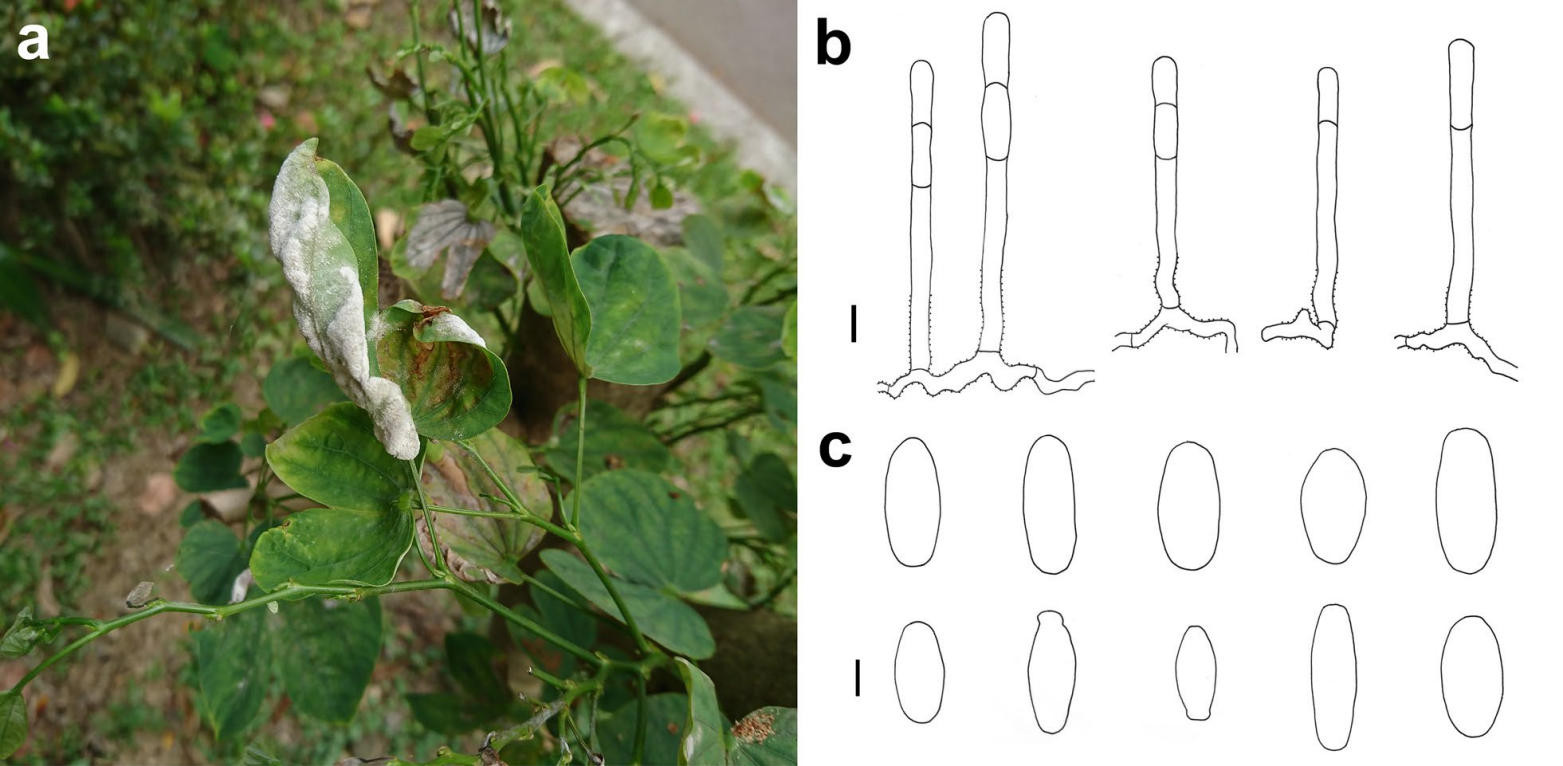
*Erysiphe lespedezae* on *Bauhinia* sp. (AKII 0055) **a** Powdery mildew symptoms on leaves. **b** Hyphae with nipple-shaped appressoria and conidiophores with curved foot cell. **c** Conidia. Scale bars = 10 μm

*Specimens examined* On leave blades and petioles of *Bauhinia* sp. (Fabaceae). Taipei City, Daan District, National Taiwan University campus, ca. 25.016553, 121.540245, ca. 15 m, 7 Apr 2019, AKII 0014 (not preserved), ITS sequence GenBank MW290427; same place, 09 Apr 2019, R. Kirschner 4714 (TNM); same place, 26 Feb 2020, AKII 0055 (TNM); Hualien County, Shoufeng Township, National Dong-Hua University, ca. 23.897224, 121.542145, ca. 45 m, 3 Mar 2020, leg. R. Kirschner, AKII 0061 (TNM), ITS sequence GenBank MW290428. On leaves of *B. variegata* L., Taichung City, Wufeng District, close to Taiwan Provincial Consultative Council, ca. 24.054392, 120.701111, ca. 100 m, 11 May 2013, leg. R. Kirschner, LWA 17A (TNM).

*Notes* When ITS sequences exceeding 600 bp were compared, there was 98% to 100% identity with 1 to 7 different bp between our specimens and published specimens labeled as *E. lespedezae* in GenBank, whereas the identity with other species was 96% or lower, with more than 4 bp different. In a previous study (Liu and Kirschner 2013), we identified a specimen on *B. variegata* as *Pseudoidium caesalpiniacearum* (Hosag. & U. Braun) U. Braun & R.T.A. Cook because of the long foot cell of the conidiophore. Following the species concept of *E. lespedezae* on *Bauhinia* and other hosts in Meeboon and Takamatus (2017a), however, distinction between these species based on the foot cell can no longer be upheld. The sizes of the foot cells varied also considerably between our specimens on different hosts (see below).

### *Erysiphe lespedezae* R.Y. Zheng & U. Braun on *Desmodium caudatum* Fig. 6

*Colonies amphigenous on leaves* Hyphae smooth, 2–5 μm wide. Hyphal appressoria solitary or in opposite pairs, nipple-shaped or lobed. Conidiophores arising solitarily from middle of hyphal mother cell, slightly curved and rough at base, composed of foot cell and 1–3 distal cells. Foot cell with basal septum at same level as upper surface of supporting hypha, straight or curved, (20–)24–35(–40) × 5–6(–7) μm. Conidia solitary, ellipsoid-ovoid to doliiform, (27–)30–38(–41) × (12–)13–15(–17) μm, germination close to one end with short hypha.

**Fig. 6:**
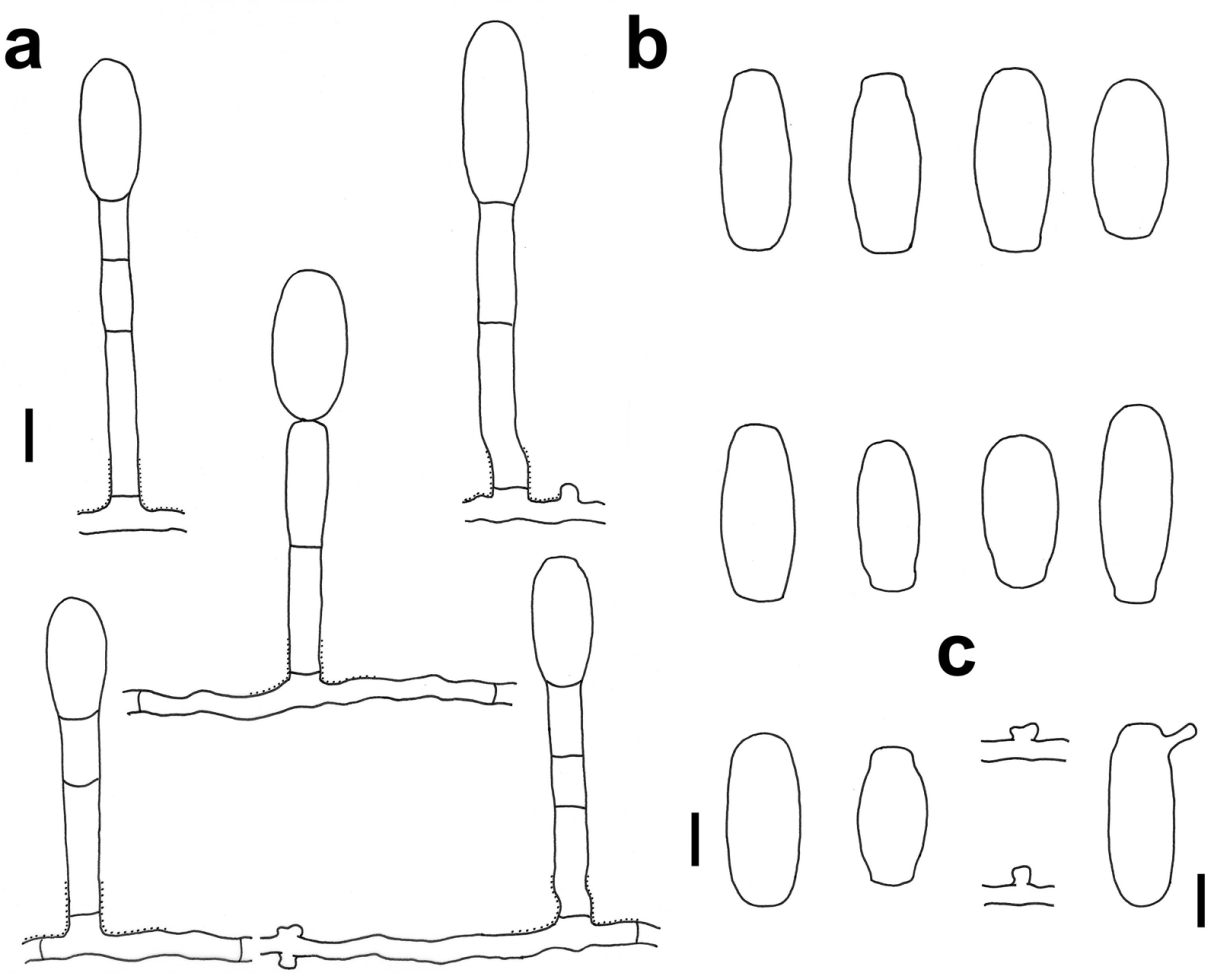
*Erysiphe lespedezae* on *Desmodium caudatum* (R. Kirschner 4646). **a** Hyphae with lobed appressoria and conidiophores. **b** Conidia. **c** Conidia with germination hyphae and appressoria

*Specimens examined* On leaves of *Desmodium caudatum* (Thunb.) DC. (Fabaceae). Taoyuan City, Zhongli District, plant market, ca. 24.956456, 121.218250, ca. 130 m, 7 Jul 2018, R. Kirschner 4646 (TNM), ITS sequence GenBank MW290429.

*Notes* A powdery mildew was reported on *D. caudatum* in Taiwan, but not further identified (Hung 2016). The fungus was identified based on our previous collection from Yingge, New Taipei City (Wen 2019), but the material was not preserved.

### *Erysiphe lespedezae* R.Y. Zheng & U. Braun on *Uraria crinita* Fig. 7

*Colonies amphigenous on leaves* Hyphae smooth, 3–6 μm wide. Hyphal appressoria mostly solitary, nipple-shaped or lobed. Conidiophores arising solitarily from middle of hyphal mother cell, slightly curved and rough at base, composed of foot cell and 1–4 distal cells. Foot cell with basal septum at same level as upper surface of supporting hypha, straight or curved, (35–)45–78(–115) × (4–)5–6(–8) μm. Conidia solitary, ellipsoid-ovoid to doliiform, smooth-walled, (24–)26–34(–40) × (12–)14–17(–19) μm, germination terminal.

**Fig. 7:**
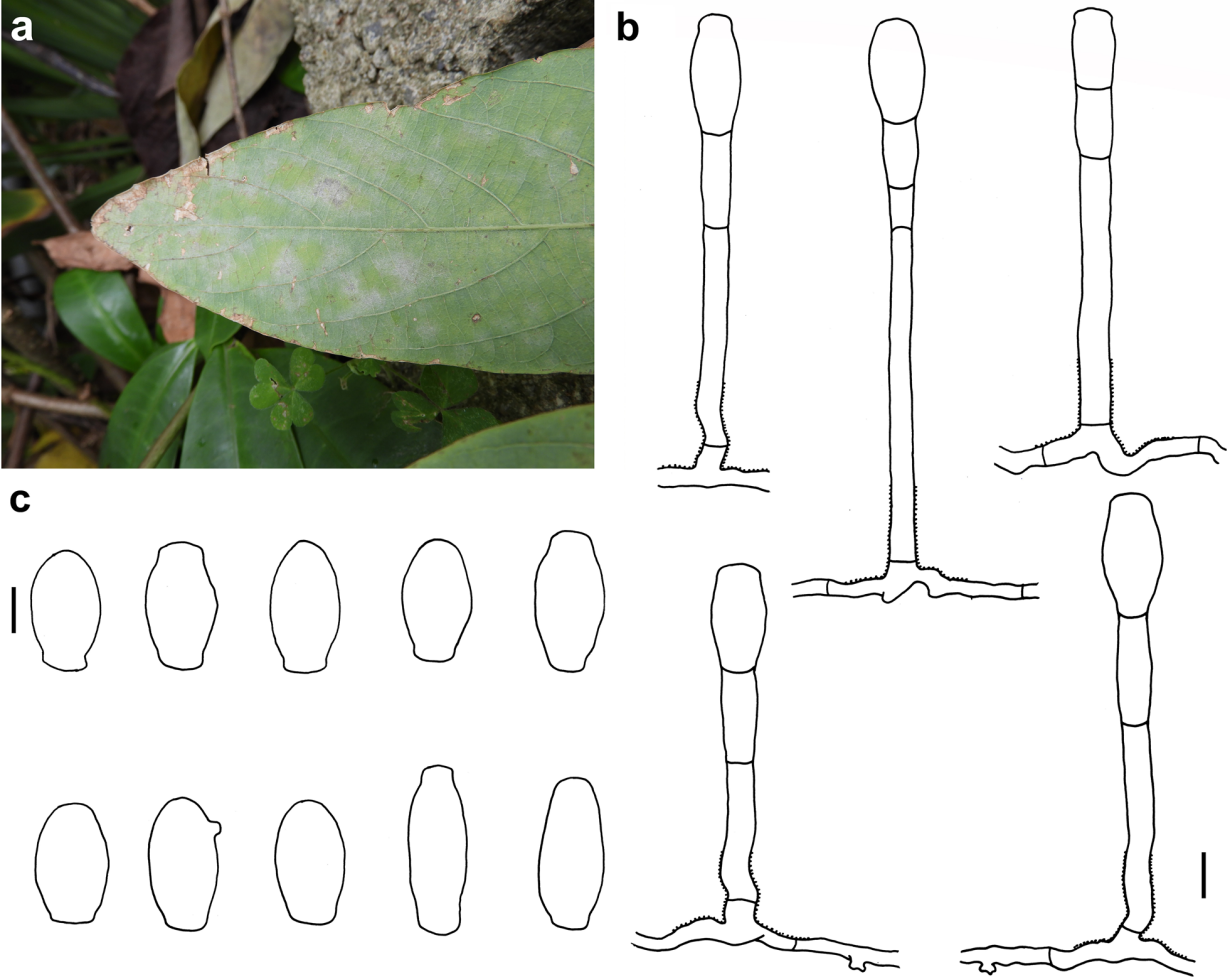
*Erysiphe lespedezae* on *Uraria crinita* (AKII 0005). **a**. Powdery mildew symptoms on leaf. **b** Hyphae with lobed appressoria and conidiophores with curved foot cell. **c** Conidia. Scale bars = 10 μm

*Specimens examined* On leaves of *Uraria crinita* (L.) Desv. ex DC. (Fabaceae). Miaoli County, Touwu Township, Lavender Cottage, ca. 24.587072, 120.896818, ca. 80 m, 09 Nov 2018, AKII 0005 (TNM), ITS sequence GenBank MT703848; Taipei City, Shilin District, Medicinal Botanical Garden, 25.115751, 121.576885, ca. 220 m, 12 Nov 2018, AKII 0006 (TNM).

*Notes Uraria* species have not yet been recorded as hosts of powdery mildews worldwide (Braun and Cook 2012; Farr and Rossman 2020). The foot cell of conidiophores was longer [(35–)45–78(–115) μm] than reported in Braun and Cook (2012): about 20–40 μm long), but Meeboon and Takamatus (2017a) also reported longer foot cells (35–65 μm) for this species. Some species of Erysiphales on Fabaceae presently lacking DNA, such as *Pseudoidium bauhiniae* (G.J.M. Gorter & Eicker) U. Braun & R.T.A. Cook and *Ps. caesalpiniacearum* (Hosag. & U. Braun) U. Braun & R.T.A. Cook may require restudy for clarifying the species boundaries of *E. lespedezae*.

### *Erysiphe lonicerae* DC. Fig. 8

*Colonies amphigenous on green leaves* Hyphae verruculose, 3–6 μm wide. Hyphal appressoria mostly single, rarely in opposite pairs, nipple-shaped, lobed or elongated. Conidiophores arising solitarily from middle of hyphal mother cell, straight or curved at base (only in one case curvature formed in cell distal to foot cell), cylindrico-clavate, verruculose, composed of foot cell and 1–3 distal cells, (55–)79–120(–140) × (7–)9–12(–14) μm. Foot cell with basal septum at same level as upper surface of supporting hypha, cylindrical, straight or curved, (20–)37–51(–60) × 5–6.5(–7) μm. Conidia solitary, oblong to short cylindrical, smooth-walled when fully turgescent, wall becoming reticulate by aging, (31–)32–40(–44) × (13–)15–18.5(–20) μm, germinating apically into a short hypha with lobed appressoria.

**Fig. 8:**
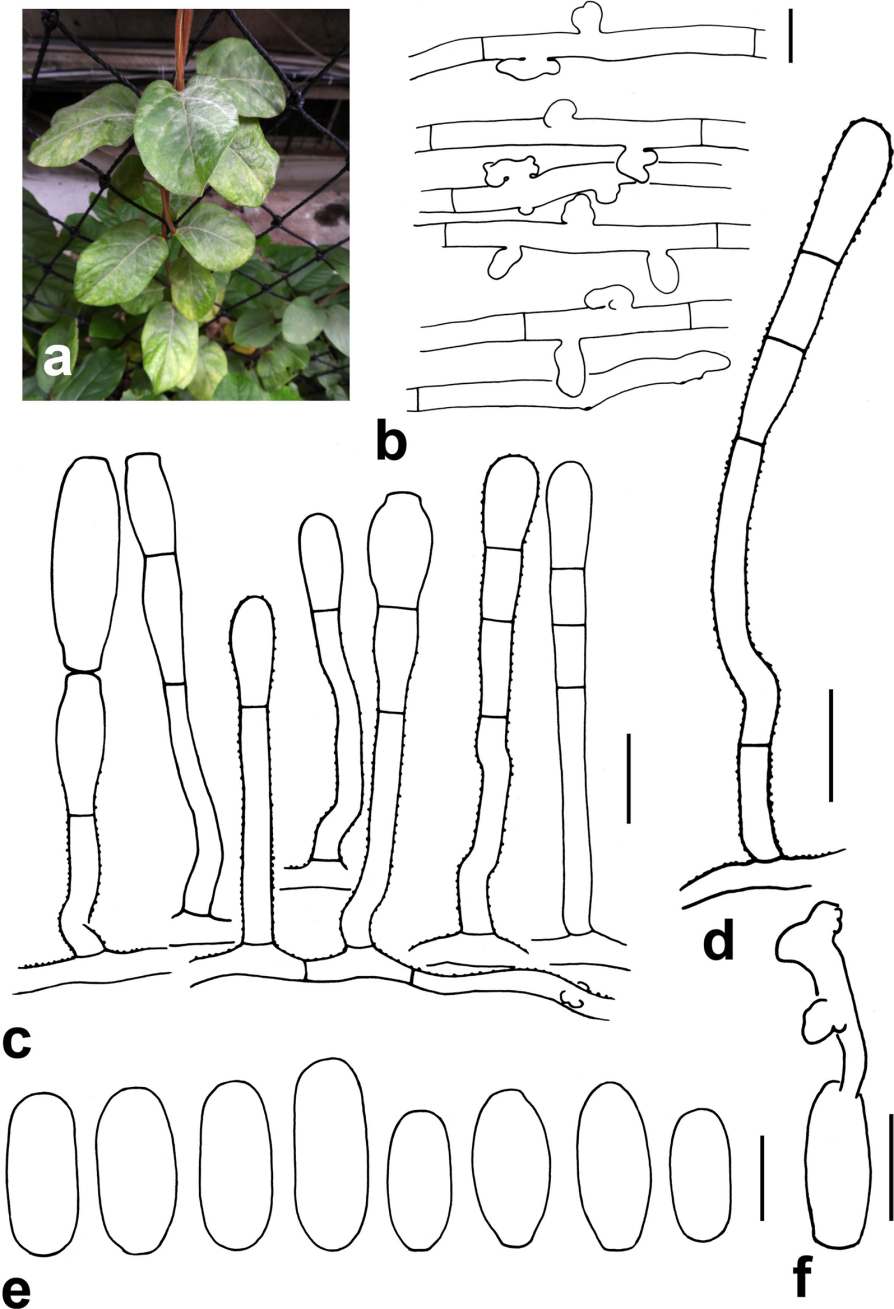
*Erysiphe lonicerae* on *Lonicera japonica* (R. Kirschner 4712, except a = R. Kirschner 4733). **a** Powdery mildew symptoms on leaves. **b** Hyphae with appressoria. **c** Conidiophores with straight or curved foot cell (surface ornament not shown in all conidiophores). **d** Exceptional conidiophore with curvature above foot cell. **e** Conidia. **f** Conidium with appressoria on the germination hypha. Scale bars b = 10 μm, all others = 20 μm

*Specimens examined* On mature leaves of *Lonicera japonica* Thunb. (Caprifoliaceae). New Taipei City, Shenkeng District, near City Highway 106B, ca. 24.999115, 121.620617, ca. 50 m, 07 Apr 2019, R. Kirschner 4712 (TNM), ITS sequence GenBank MT703799; Taipei City, Daan District, National Taiwan University, near post office, ca. 25.018563, 121.537213, ca. 15 m, 31 May 2019, R. Kirschner 4733 (TNM); Taipei City, Shilin District, Medicinal Botanical Garden, 25.115751, 121.576885, ca. 220 m, 30 Oct 2014, R. Kirschner & W.-A. Liu 4112 (TNM).

*Notes* When ITS sequences exceeding 600 bp were compared, there was 99% identity with 1 to 4 different bp between our specimen and seven published specimens labeled as *E. lonicerae* in GenBank, whereas the identity with other species was 98% or lower, with more than 10 different bp. *Erysiphe lonicerae* is known on different *Lonicera* species in Europe and East Asia (mainland China, Korea, Japan) and is a new record for Taiwan (Farr and Rossman 2020). Information about conidiophore morphology and DNA sequences do not exist, however, for two other *Erysiphe* species on *Lonicera* hosts in East Asia, namely *E. lonicerae*-*ramosissimae* (Tanda) U. Braun & Takam. and *E. miurae* (U. Braun) U. Braun & Takam. These data are also lacking for *E. magnusii* (S. Blumer) U. Braun & Takam. reported from Europe and Central Asia, and for *E. caprifoliacearum* (U. Braun) U. Braun & Takam. in America (Braun and Cook 2012).

## Discussion

### Advances and limitations of molecular species recognition

Identification of powdery mildew fungi to species can be based on the traditional combination of host specificity and fungus morphology, particularly of the sexual stage, when sufficient data are available in a given area. Since the sexual stage is rarely found in the tropical/subtropical environment in Taiwan and data are far from being sufficient for Erysiphales in Taiwan, ITS sequence-based identification is an important requirement for species identification of the powdery mildews. For obtaining DNA from powdery mildews, careful selection of clean, fresh specimens is necessary, since powdery mildews cannot be cultivated in vitro, are often contaminated by other microscopic fungi and DNA deteriorates quickly during drying specimens. In Erysiphales, the ITS is the single available barcode for species identification particularly through the accomplishments of Takamatsu (Takamatsu et al. 2015a, b; Meeboon and Takamatus 2016, 2017a, b, 2020). Five species of powdery mildews were identified here based primarily on ITS sequence data. The fungus recorded by Sawada (1959) as *E. pisi* DC. on *Lespedeza bicolor* in Taiwan, may actually be *E. lespedezae*. This is an example for using ITS sequence data for progress in former problematic identifications. In Erysiphales, the barcode gap of ITS sequences is often very narrow, which was also confirmed in our study with 99 to 100% sequence identities and a variation of 0 to 6 different bp found in the same species. The variation depends on the available data as well as the species, since, for example 6 different bp were still within the range of variability in *E. heraclei*, whereas for *E. lespedezae* the same number of different bp would indicate an overlap with other, closely related species. Because of the high identities between ITS sequences of related species, even in phylogenetic analyses it is sometimes to not possible to resolve the species which then still remain provisionally based on the traditional combination of host occurrence and fungal morphology (Chen and Kirschner 2018). In the case of *E. glycines* and *E. lespedezae*, both species are treated as synonyms of *E. diffusa* (Cooke & Peck) U. Braun & S. Takam. in Index Fungorum, but are separated in Braun and Cook (2012). The two fairly well supported subclades in each of both clades indicate the presence of further two more species (Fig. 1). In *E. glycines*, the well-supported subclade formed by the two specimens on *Pueraria lobata* may represent *E. puerariae*, which is hitherto only known from *P. lobata* in mainland China and for which hitherto no anamorph or DNA data are available.

Since in the clade of *E. lespedezae* we could presently not find a correlation between distinct morphological characteristics such as the length of foot cell of the conidiophore or host specificity (e.g. *Bauhinia* hosts occur in both subclades), we preliminarily adopt the present broad species concept of *E. lespedezae* (Meeboon and Takamatsu 2017). In the future, perhaps one of the anamorph names for powdery mildews on *Bauhinia* hosts (see above) may reveal to be applicable to one of those subclades. In the example of *E. lonicerae*, we demonstrate that for several species on the same host genus, information of DNA data and anamorph morphology is lacking. *Erysiphe heraclei* is an example for illustrating the conflict between the ITS phylogeny and traditional taxonomy. Most recent new attempts to obtain DNA from herbarium specimens of Erysiphales and to develop alternative barcodes for this group of fungi are very promising for resolving these problems (Ellingham et al. 2019; Bradshaw and Tobin 2020).

### Origin of host plants and detection of associated fungi

Except for *L. chinense*, the medicinal plants of this study are also valued as ornamentals (Hsueh and Yang 2011, 2014, 2016). Among these plants cultivated in Taiwan, *D. caudatum*, *Lonicera japonica* and *U. crinita* are native (Ohashi and Iokawa 2007). The parasitic fungi on these hosts may be native, but have escaped attention due to limited research of fungi. *Lycium chinense* was introduced from mainland China ca. 300 years ago (Wu et al. 2010), *Arthrocladiella mougeotii* has been introduced on its *Lycium* hosts from Eurasia to other countries and continents (Braun and Cook 2012) and also most likely from mainland China to Taiwan. *Eryngium foetidum* was introduced to Taiwan in 1926 (Wu et al. 2010). *Bauhinia* species were also introduced in the 20th century for ornamental purposes (Wu et al. 2010). *Bauhinia* spp., *L. chinense* and *E. foetidum* have been classified as medicinal plants as well as potential weeds (Kumar and Chandrashekar 2011, Singh et al. 2019, Wu et al. 2010). The kudzu (*P. lobata*) is a native weed, which has become invasive in other countries so that pathogens may provide some potential for biological control (Kirschner et al. 2013). In contrast to another pathogen on kudzu leaves, *Marasmius puerariae* R. Kirschner (Kirschner et al. 2013), the powdery mildew *E. glycines* in its present broad species concept (see above) is not specific to kudzu, but can also infect economically valuable crops among the Fabaceae. Even if this powdery mildew on kudzu may be classified as more kudzu-specific species, the potential for control of kudzu would be low because of the rarity of the pathogen on kudzu.

In some plant parasitic fungi, such as smut fungi (Basidiomycota, Ustilaginomycotina), it is possible to retrieve new information of fungal distribution from phanerogam herbarium specimens (Piepenbring 2001). Our scanning of over 80 herbarium specimens of the four host plants in TAI for the accidental presence of powdery mildews, however, did not retrieve any fungi except for few saprobic molds. It is, therefore, not possible to conclude from plant specimens whether associated powdery mildews have escaped from attention in the past or have been introduced from abroad more recently. This result also shows the importance of specific mycological field study in order to complement the insufficient data of plant pathogenic fungi as well as of building up and curating professional mycological specimen collections.

## Conclusions

Five species of powdery mildews on medicinal plants as new hosts from Taiwan were identified here based primarily on ITS sequence data. Our new records and the lack of DNA data as well as anamorph characteristic in several *Erysiphe* species show that knowledge of Erysiphales is far from being complete. Compared to plant biogeography, mycological exploration apparently suffers from more limitations concerning funding and numbers of researchers. Such study is relevant for estimating risks for quarantine and for growing medicinal plants under particular strategies of crop marketing.
